# Effective treatment of osmotic demyelination syndrome with plasmapheresis: a case report and review of the literature

**DOI:** 10.1186/s13256-020-02573-9

**Published:** 2021-01-11

**Authors:** Maheshi Wijayabandara, Shenal Appuhamy, Praveen Weerathunga, Thashi Chang

**Affiliations:** 1 University Medical Unit, National Hospital of Sri Lanka, Colombo, Sri Lanka; 2 Department of Clinical Medicine, Faculty of Medicine, University of Colombo, Colombo, Sri Lanka

**Keywords:** Hyponatraemia, Osmotic demyelination, Plasmapheresis, Sri Lanka

## Abstract

**Background:**

Treatment options for chronic osmotic demyelination syndrome are limited to case reports and only a very few show complete recovery. We report a case of complete recovery of chronic osmotic demyelination syndrome with plasmapheresis.

**Case presentation:**

A 43-year-old Sri Lankan man presented with fever, repeated vomiting, unsteady gait, increased tonicity of his right upper limb and paucity of speech for three days. He was treated in the local hospital with antibiotics and antivirals as per central nervous system infection. He had hyponatraemia, which was rapidly corrected with hypertonic saline from 97 to 119 mmol/L. He was transferred to our hospital because of progressive reduction of consciousness, rapidly worsening rigidity and bradykinesia of all four limbs and worsening dysarthria and bradyphrenia. Magnetic resonance imaging of the brain was compatible with osmotic demyelination syndrome. He was commenced on plasmapheresis twenty-two days after rapid correction of sodium. He regained independent mobility with complete resolution of rigidity, bradykinesia and speech dysfunction after five cycles of alternate day plasmapheresis.

**Conclusion:**

Plasmapheresis can be considered as an effective treatment modality in chronic osmotic demyelination syndrome.

## Background

Rapid correction of hyponatraemia is associated with osmotic demyelination syndrome (ODS), which is a demyelinating disorder of the central nervous system (CNS) [1]. Brain adapts to chronic hyponatraemia by extracellular movement of osmotically active organic and inorganic particles [2]. During rapid correction of hyponatraemia, organic osmolytes cannot re-enter the intracellular compartment as rapidly as ionic movement creating an osmotic disequilibrium [2]. This causes shrinkage of brain cells including astrocytes and oligodendrocytes causing accelerated apoptosis leading to disruption of the blood brain barrier and demyelination [3]. Consequently, symptoms related to pontine and extra-pontine demyelination typically occurs after two to six days of rapid sodium correction [4]. Traditionally, established ODS is considered to be associated with a poor prognosis [4]. Apart from supportive therapy, sodium re-lowering therapy has shown to be beneficial in the acute stage [5]. However, successful outcome of chronic ODS is limited to a few case reports [6, 7]. Out of the experimental therapies, plasmapheresis alone or in combination with other treatment modalities has shown variable benefit [6, 7, 8, 9, 10, 11]. We report a case of complete clinical and radiological recovery of ODS with plasmapheresis, initiated twenty-two days after rapid sodium correction.

## Case presentation

A 43-year-old Sri Lankan man with type 2 diabetes mellitus and hypertension presented with fever for 3 days which was associated with arthralgia, myalgia, dry cough and headache, but without features of meningism. Fever resolved after 3 days and he was well except for arthralgia and myalgia. After 1 week of resolution of fever he developed recurrent episodes of vomiting followed by development of an unsteady gait with increased tonicity of his right upper limb and paucity of speech. There was no history of altered level of consciousness, involuntary movements or seizures. He was admitted to the local hospital with above symptoms on the 12th day of his illness. On admission to the local hospital he was afebrile, pulse rate was 104 bpm and blood pressure was 130/80 mmHg. His Glasgow coma scale score (GCS) was 15/15, pupils were equally reactive to light and there was no neck stiffness. During the hospital stay he had resurgence of fever with worsening rigidity and difficulty in walking. He was noted to have a sodium of 97 mmol/L, which was corrected with hypertonic saline up to 119 mmol/L. His haematological and biochemical investigations done at local hospital are shown in Table 1. After 4 days of sodium correction he developed reduced level of consciousness, bradykinesia and tremors of both upper and lower limbs symmetrically. His speech remained sparse and there were no seizures. Non contrast computed tomography (NCCT) brain was normal. Cerebro-spinal fluid (CSF) analysis was not done. He was transferred to the National Hospital of Sri Lanka (NHSL), Colombo on the 22nd day of his illness for further management.

**Table 1.** Haematological and biochemical parameters of the patient at the local hospital.

Laboratory parameter	Value	Reference range
Haematology		
Total white cell count (cells/µL)	17,140	4000–11,000
Neutrophil count (cells/µL)	13,026	1500–8000
Lymphocyte count (cells/µL)	2802	1000–4800
Haemoglobin level (g/dL)	14.2	13.5–17.5
Platelet count (platelets/µL)	352,000	150,000–450,000
Erythrocyte sedimentation rate (mm/1st hour)	6	
Biochemistry		
C-reactive protein (mg/L)	28	< 6
Serum creatinine (µmol/L)	98	60–110
Serum potassium (mmol/L)	4.1	3.5–5.1
Serum osmolality (mOsm/kg)	214	275–295
Urine osmolality (mOsm/kg)	632	300–900
Urine sodium (mmol/L)	51	< 20
Serum 9 am cortisol (nmol/L)	380	> 300
Serum thyroid stimulating hormone (mIU/L)	1.16	0.4–4

There was no history of recent travel, animal contact, familial movement disorders, past history of psychiatric disorders or exposure to toxic substances including methanol, ethylene glycol or cyanide. He was a non-smoker and consumed alcohol occasionally at social events.

On examination, his body mass index was 24.2 kg/m^2^, afebrile and there was no neck stiffness. GCS was 11/15 (best motor reponse—6, best verbal response—2, and eye opening—3). He had features of Parkinsonism including bradykinesia, rigidity and tremors symmetrically involving both upper and lower limbs. Increased muscle tone and hyper-reflexia were noted in all four limbs with bilateral extensor plantar responses. He had ignition failure and a narrow-based shuffling gait. Speech was sparse with an expressionless face (see Additional file 1 for video 1). Cardiovascular, respiratory and abdominal examinations were normal.

His haematological and biochemical investigations done at NHSL are shown in Table 2.

**Table 2.** Haematological and biochemical parameters of the patient at National Hospital of Sri Lanka

Laboratory parameter	Value	Reference range
Haematology		
Total white cell count (cells/µL)	9820	4000–11,000
Neutrophil count (cells/µL)	7430	1500–8000
Lymphocyte count (cells/µL)	1550	1000–4800
Haemoglobin level (g/dL)	12.9	13.5–17.5
Platelet count (platelets/µL)	319,000	150,000–450,000
Erythrocyte sedimentation rate (mm/1st hour)	6	
Biochemistry		
C-reactive protein (mg/L)	38	< 6
Serum creatinine (µmol/L)	88	60–110
Serum sodium (mmol/L)	138	135–148
Serum potassium (mmol/L)	3.99	3.5–5.1
Creatine phosphokinase (U/L)	123	39–308
Aspartate aminotransferase (U/L)	31	10–40
Alanine aminotransferase (U/L)	23	7–56
Alkaline phosphatase (U/L)	66	53–128

**Fig. 1:**
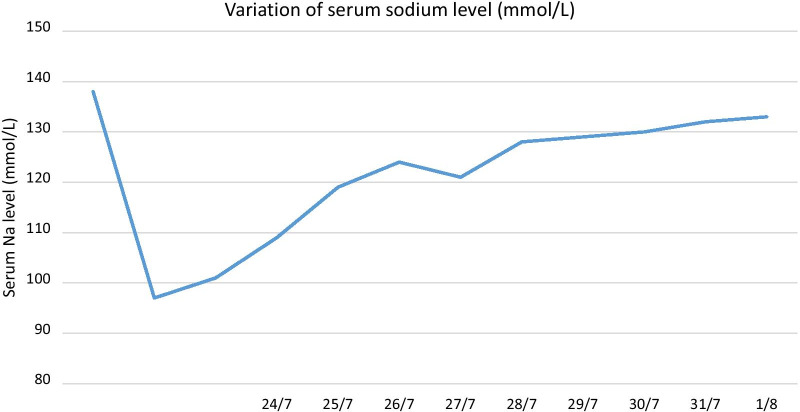
Temporal variation of serum sodium levels. The graph shows rapid sodium correction from a trough of 97 to 119 mmol/L.

**Fig. 2:**
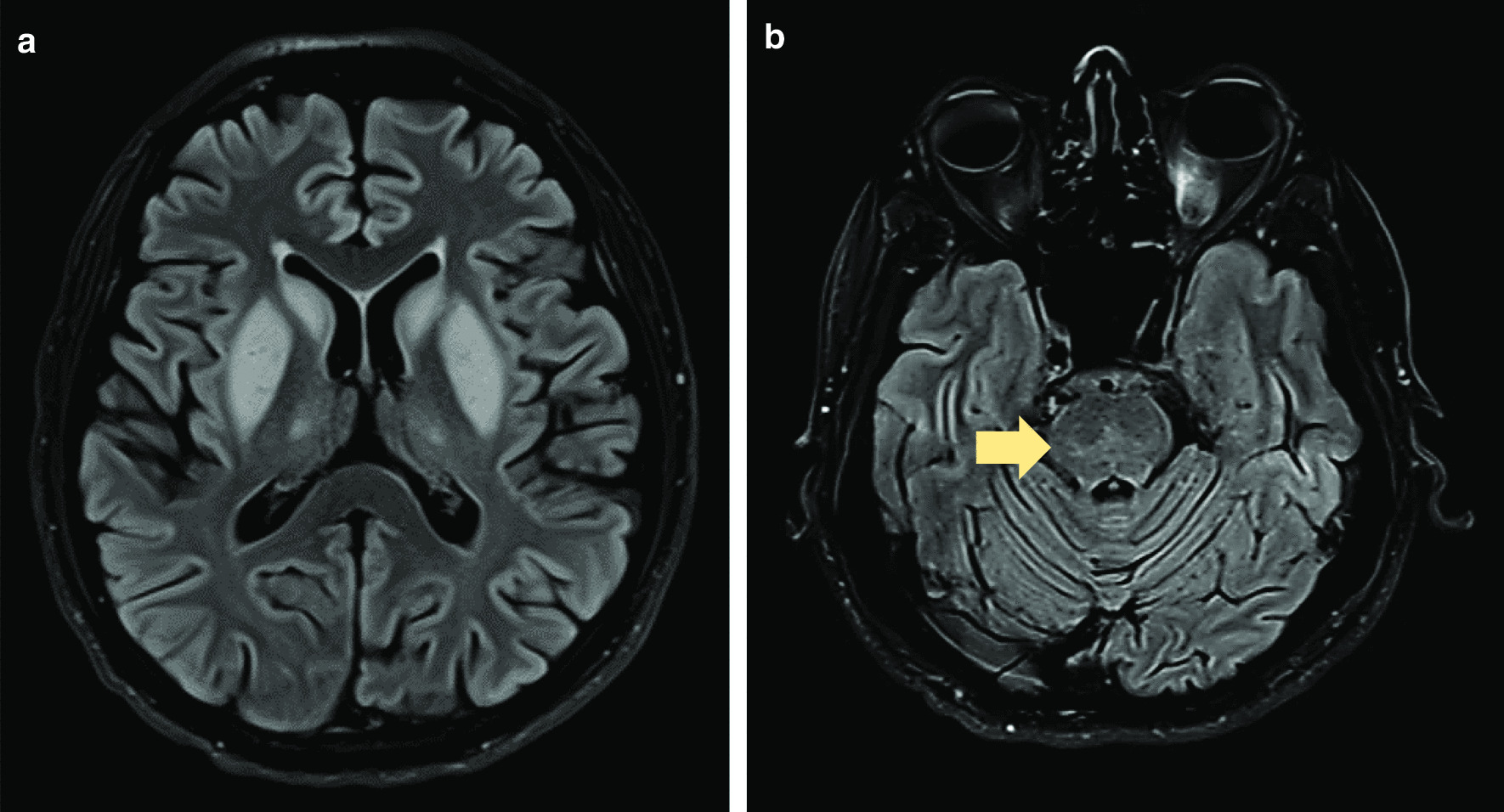
MRI brain before plasmapheresis. **a** Bilateral symmetrical T2-FLAIR high signal involving caudate, lentiform nuclei, thalami and external capsules; **b** central trident shaped T2-FLAIR high signal area in the pons (yellow arrow).

Screening for sepsis including urine full report, urine/ blood cultures and chest radiograph were unremarkable. Variation of his serum sodium levels are shown in the Fig. 1. Sodium level was 138 mmol/L on admission to our hospital. NCCT brain was normal. CSF analysis on the 23^rd^ day of the illness, was normal except for an elevated level of protein. Antibodies against Japanese encephalitis, polymerase chain reaction of *Herpes simplex virus* and *Mycobacterium tuberculosis* were negative. Magnetic resonance imaging (MRI) of the brain showed bilateral symmetrical T2 FLAIR (fluid-attenuated inversion recovery) high signal involving caudate, lentiform nuclei, thalami and external capsules. Furthermore, the characteristic central trident shaped T2 FLAIR high signal area was evident in the pons (Fig. 2). A diagnosis of ODS was made based on the history of rapid sodium correction and abnormalities detected on the cranial MRI.

**Fig. 3:**
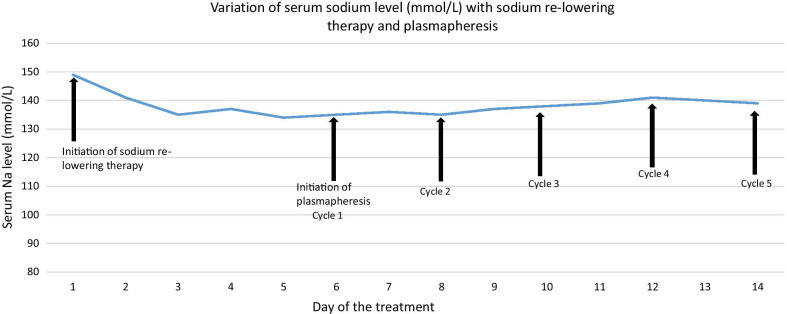
Temporal variation of serum sodium levels with sodium re-lowering therapy and plasmapheresis.

**Fig. 4:**
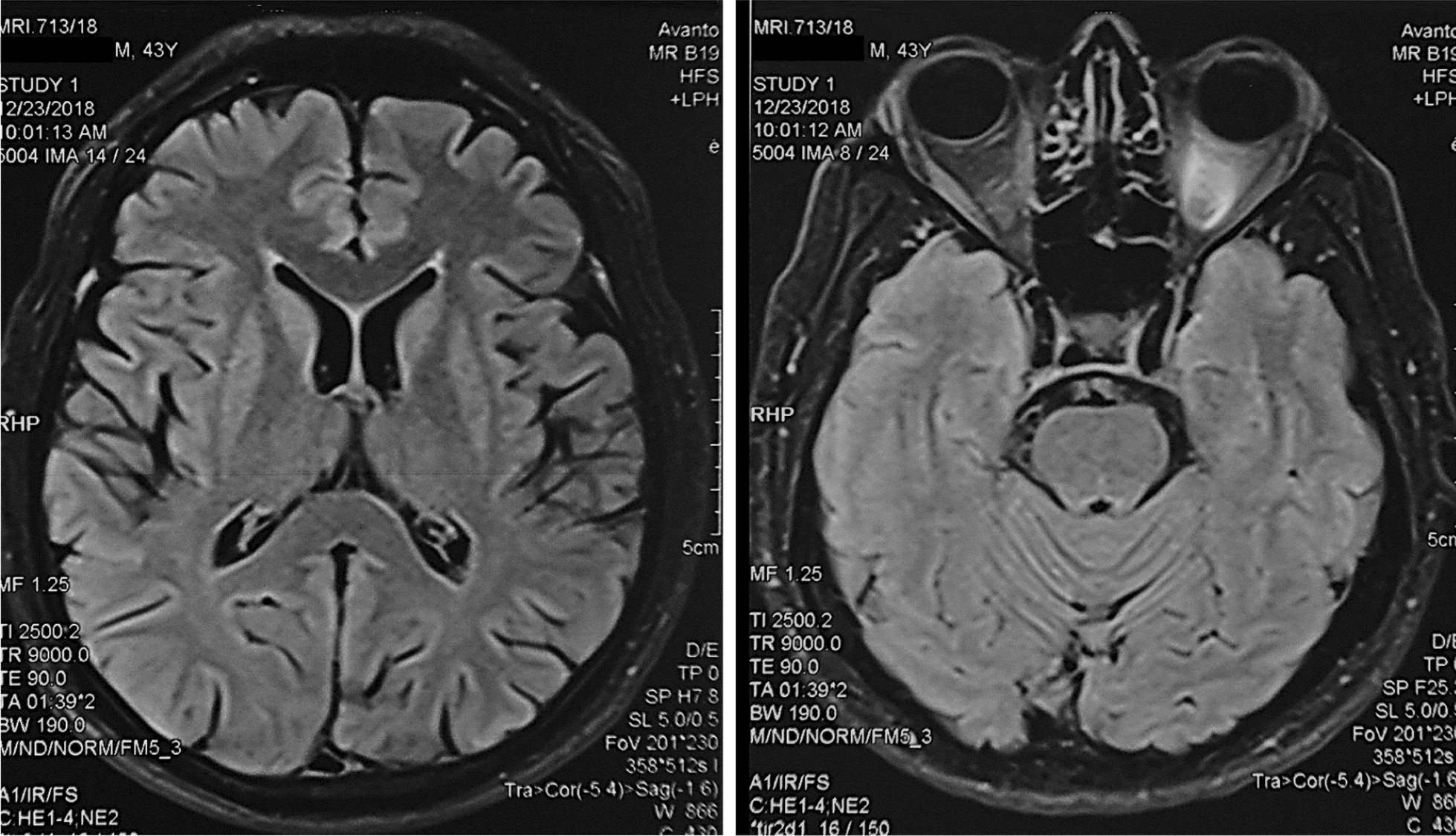
MRI brain five months after plasmapheresis. The images shows complete resolution of changes observed in Fig. 2.

As infection in the CNS was suspected intravenous ceftriaxone and aciclovir were administered for fourteen days. Parkinsonism was treated symptomatically with co-careldopa and benzhexol. Intubation was not needed and he was fed via a naso-gastric feeding tube. In order to reverse the effects of ODS, sodium re-lowering therapy was attempted with 5% dextrose but failed to achieve clinical improvement and induce target hyponatraemia. Thus, the patient was initiated on alternate day plasmapheresis with a total plasma exchange volume of 11,132 mL. There was improvement of his rigidity, tremors and bradykinesia with plasmapheresis but without improvement in his speech and gait initially. However, at the end of five cycles there was complete neurological recovery (see Additional file 2 for video 2). Variation of sodium level with sodium re-lowering therapy and plasmapheresis is shown in Fig. 3. Neurological reassessment done three months after discharge showed sustained complete recovery and repeat MRI brain showed complete resolution at five months (Fig. 4; Additional file 3: Timeline).

## Discussion

We report a case of chronic ODS that showed complete clinical and radiological resolution with plasmapheresis. The initial clinical presentation of our patient to the local hospital with fever and repeated vomiting followed by unsteady gait, increased tone of the right upper limb and paucity of speech suggested a CNS infection involving the basal ganglia. This clinical presentation was consistent with a CNS infection such as acute encephalitis with predilection to basal ganglia. Flaviviruses such as Japanese encephalitis and West Nile virus are known to cause encephalitis with basal ganglia involvement, although a number of other viruses, toxoplasmosis and tuberculosis may also cause a similar syndrome. [12, 13].

The cause of the hyponatraemia detected at the local hospital was probably multifactorial, contributed by sodium loss through vomiting and syndrome of inappropriate anti-diuretic hormone secretion due to CNS infection. This had been rapidly corrected during the hospital stay. Rapid correction of hyponatraemia is known to cause ODS [1]. The clinical course with predominant extrapyramidal symptoms could be explained either by the progressive CNS infection or concurrent development of ODS. However, T2 FLAIR MRI images showing symmetrical high intensity lesions in the caudate, lentiform nuclei and bilateral thalami with sparing of globus pallidus and the typical trident shaped T2 high intensity lesion in the pons favour ODS [14].

Apart from supportive care, there is no proven treatment for established ODS. Re-lowering of sodium using 5% dextrose and desmopressin have shown benefit in treating ODS in animal models, but data in humans is limited to case reports and case series [5, 15]. However, successful reversal of neurological manifestations had only being achieved with early re-induction of hyponatraemia. Although sodium re-lowering was initially started in our patient to treat ODS, it was soon discontinued due to the lack of any clinical improvement. Other treatments modalities reported for ODS included administration of thyrotrophic releasing hormone, corticosteroids [16], immunoglobulins [17] and plasmapheresis. These had been used alone or in combination and the outcomes had been variable [11].

Plasmapheresis is an extracorporeal treatment aimed to remove circulating pathologic or toxic substances from the circulation, in order to halt the progression of a disease process. There are several case reports describing plasmapheresis used alone or in combination with other treatment modalities but the reported outcomes have been variable (Table 3).

**Table 3.** Summary of case reports describing plasmapheresis used to treat ODS.

Age and gender of the patient	Clinical features	MRI findings		Day of initiation of plasmapheresis	No. of cycles/volume exchanged	Outcome		References
		Pontine	Extra-pontine			Clinical	Radiological	
29 F	Tetraparesis, areflexia, bulbar dysfunction	+	−	NA	24,700 mL	Partial	Unchanged	[8]
20 F	Spastic tetraparesis, bulbar dysfunction	+	−	NA	5243 mL	Partial	Unchanged	[8]
30 F	Tetraparesis, pseudo-bulbar palsy, nystagmus	+	−	NA	18,270 mL	Complete	Unchanged	[8]
59 F	Pseudo-bulbar palsy, flaccid tetraplegia, ophthalmoplegia, hyper-reflexia	+	−	A week after the onset of neurological manifestations	37,300 mL	Partial	Unchanged	[9]
40 F	Tetraparesis, pseudo-bulbar palsy, diplopia	+	−	Immediately after MRI confirmation of ODS	4394 mL	Partial	NA	[10]
71 F	Spastic tetraparesis, hyper-reflexia, dysphagia	+	+	23rd day after rapid sodium correction	3840 mL	Complete	The signals were reduced in size but remained	[6]
50 F	Drowsy, spastic tetraparesis, areflexia, bulbar dysfunction	+	−	20th day after the onset of neurological manifestations	7 cycles	Complete	NA	(7)

*F* female, *NA* data not available, + presence of changes, − absence of changes

The pathogenesis of ODS is incompletely understood. It is believed that chronic hyponatraemia is associated with movement of osmotically active particles out in to the extracellular compartment to prevent swelling of astrocytes. However, when the hyponatraemia is rapidly corrected, organic osmolytes cannot re-enter to maintain intracellular osmolarity, as fast as the extracellular osmolarity is increased by infusion of sodium. This results in shrinkage of astrocytes and oligodendrocytes resulting in their apoptosis, inflammation and disruption of blood-brain barrier leading to demyelination [2, 18]. Hence, brain areas which have slowest uptake of osmolytes such as central pons (30–50%), extra-pontine areas (20–50%) or both (30–50%) are the worst affected [19]. There is also evidence for the role of an undefined myelinotoxin released due to osmotic stress contributing to myelinolysis [8].

An unknown myelinotoxic substance in plasma has been postulated to mediate ODS [6]. This substance is thought to gain access into the CNS by damage to the blood brain barrier and is the possible mechanism of prolonged and ongoing neurotoxicity [6]. Efficacy of plasmapheresis in ODS is thought to be related to removal of pro-inflammatory, high molecular weight myelinotoxins from the circulation [8].

Chronic established ODS was traditionally considered to have a poor neurological outcome. Mortality associated with ODS has been reported in up to 31% of patients while another 31% have been reported to require lifelong supportive therapy [20]. In contrast, our case report highlights the benefit of plasmapheresis in established ODS even when initiated later in the course of the disease.

## Conclusion

Although chronic established ODS has been considered to be associated with a poor outcome, this case report highlights that plasmapheresis may remain effective in reversing ODS several weeks after the initial osmotic insult.

## Supplementary information

**Additional file 1:** **Video 1.** Demonstration of clinical features of the patient before initiating plasmapheresis.

**Additional file 2:** **Video 2.** Demonstration of complete neurological recovery of the patient at the end of plasmapheresis.

**Additional file 3:** **Timeline.** Summary of the important clinical events of the patient shown in a timeline.

## Data Availability

All necessary data and material are provided.

## Abbreviations

ODS: Osmotic demyelination syndrome
CNS: Central nervous system
GCS: Glasgow coma scale
NHSL: National Hospital of Sri Lanka
CSF: Cerebrospinal fluid
MRI: Magnetic resonance imaging
FLAIR: Fluid-attenuated inversion recovery
