# Supplementary material for: Effective treatment of osmotic demyelination syndrome with plasmapheresis: a case report and review of the literature
Source: J Med Case Rep. 2021 Jan 11;15:6. doi: 10.1186/s13256-020-02573-9 (PMC7798224; doi:10.1186/s13256-020-02573-9)
Supplement: Supplementary file 3 — Additional file 3: Timeline. Summary of the important clinical events of the patient shown in a timeline. [file 13256_2020_2573_MOESM3_ESM.docx]

**Timeline of events**

5 months

3 months

D 45

D 35

D 34

D 32

D 31

Treated as per CNS infection.

Symptomatic treatment for Parkinsonism was administered.

D 22

D 22

D 17

D 13

D 12

D 11

D 4

D 1

Repeat MRI brain showed complete resolution

Sustained complete neurological recovery

Achievement of complete neurological recovery.

Initiation of five cycles of alternate day plasmapheresis.

Discontinuation of sodium re-lowering therapy due to absence of clinical improvement

Administration of sodium re-lowering therapy.

MRI brain showed hyper-intensities in bilateral basal ganglia, thalami and pons.

Diagnosis of ODS was made.

Features of Parkinsonism, spastic tetraparesis and bradyphrenia

Admitted to National Hospital of Sri Lanka, Colombo, Sri Lanka

Reduced level of consciousness, development of slowness and tremors of all four limbs

Vomiting

Detection of low sodium- 97 mmol/L

Rapid correction of hyponatraemia

Unsteady gait, increased tonicity of right upper limb and paucity of speech.

Admitted to local hospital.

Resolution of fever

Onset of fever
